# Proteomic Analysis of Tear Film Obtained from Diabetic Dogs

**DOI:** 10.3390/ani10122416

**Published:** 2020-12-17

**Authors:** Dagmara Winiarczyk, Mateusz Winiarczyk, Stanisław Winiarczyk, Katarzyna Michalak, Łukasz Adaszek

**Affiliations:** 1 Department of Internal Diseases of Small Animals, University of Life Sciences of Lublin, 20-950 Lublin, Poland; winiarczykdm@gmail.com; 2 Department of Vitreoretinal Surgery, Medical University of Lublin, 20-950 Lublin, Poland; mateuszwiniarczyk@umlub.pl; 3 Department of Epizootiology, University of Life Sciences of Lublin, 20-950 Lublin, Poland; genp53@interia.pl (S.W.); artica@wp.pl (K.M.)

**Keywords:** gut metabolic disease, canine diabetes, proteome, tear film, tear film biomarkers, proteomic biomarkers, MALDI, 2D electrophoresis

## Abstract

**Simple Summary:**

Canine diabetes is a serious disease, which can lead to severe complications, eventually even death. Currently, all the diagnostic procedures are the invasive ones, with blood collection remaining as a golden standard for both initial diagnosis, and later follow-up. Tears can be obtained in a non-invasive manner, which makes them a perfect candidate for a screening tool in canine diabetes. In this study we aimed to analyze the protein composition of the tears collected from the healthy animals and compared it to the diabetic group. There are significant differences between these two groups, and we believe that the identified proteins hold promise as a potential diagnostic tool, which can be later on used both in clinical practice, and for better understanding of the disease.

**Abstract:**

Canine diabetes mellitus is a significant health burden, followed with numerous systemic complications, including diabetic cataracts and retinopathy, leading to blindness. Diabetes should be considered as a disease damaging all the body organs, including gastrointestinal tract, through a complex combination of vascular and metabolic pathologies, leading to impaired gut function. Tear film can be obtained in a non-invasive way, which makes it a feasible biomarker source. In this study we compared proteomic changes ongoing in tear film of diabetic dogs. The study group consisted of 15 diabetic dogs, and 13 dogs served as a control group. After obtaining tear film with Schirmer strips, we performed 2-dimensional electrophoresis, followed by Delta2D software analysis, which allowed to select statistically significant differentially expressed proteins. After their identification with MALDI-TOF (matrix assisted laser desorption and ionisation time of flight) spectrometry we found one up-regulated protein in tear film of diabetic dogs—SRC kinase signaling inhibitor 1 (SRCIN1). Eight proteins were down-regulated: phosphatidylinositol-4 kinase type 2 alpha (PI4KIIα), Pro-melanin concentrating hormone (Pro-MCH), Flotillin-1, Protein mono-ADP ribosyltransferase, GRIP and coiled coil domain containing protein 2, tetratricopeptide repeat protein 36, serpin, and Prelamin A/C. Identified proteins were analyzed by Panther Gene Ontology software, and their possible connections with diabetic etiopathology were discussed. We believe that this is the first study to target tear film proteome in canine diabetes. We believe that combined with traditional examination, the tear film proteomic analysis can be a new source of biomarkers both for clinical practice, and experimental research.

## 1. Introduction

Diabetes mellitus (DM) is a common endocrinopathy of dogs with an estimated prevalence of 0.4–1.2% [1]. Studies suggest that genetics, an immune-mediated component, and environmental factors are involved in the development of diabetes in dogs. A variant of gestational diabetes also occurs in dogs [1]. Although ocular disease is often seen in clinical practice, its overall incidence in dogs is yet unknown [2]. It affects the cornea, lens, uvea and the retina, but also the eyelids and conjunctiva [3,4,5]. Cataract formation is the most common ocular complication of canine DM [6] although ocular surface sensory neuropathy has been also reported [7]. A recent publication has reported changes in the ocular surface in the course of diabetes mellitus including reduced tear production in 15 diabetic dogs [3]. Other reports show ocular surface pathology associated with lacrimal insufficiency in the course of diabetes [8,9,10]. Some of these complications can lead to vision loss and patient discomfort, therefore timely recognition and treatment are important.

Tear film is a thin liquid layer covering the eye, being responsible for its protection, lubrication, and nutrition and contains a variety of substances, including proteins, lipids, mucins, salts, and other organic molecules [11,12,13]. Tear proteins are believed to have key roles in protecting the ocular surface from external insults, such as pathogenic infections, and promoting ocular wound healing.

Proteomics has become an important tool in biomedical and veterinary research [12,14,15,16]. Despite well-developed veterinary ophthalmology research concerning dogs, reports on molecular studies of the tear film remains sparse. In our previous study we have identified 125 proteins in the tear film of healthy dogs [16]. It should be underlined that similar research has been already successfully conducted in human patients, providing valuable data for both clinical and research applications [17,18,19]. In this study we tried to deepen our knowledge of tear film protein composition, called proteome, in diabetic dogs. The aim of the study was to identify specific biomarkers of the DM in the tear film. They could be later used for both diagnosis and treatment of dog patients.

## 2. Materials and Methods

All the dogs from an outpatient population were consecutively enrolled through the two-year period from 2017–2019 in Innovative Center for Animal Pathology and Therapy Faculty of Veterinary Medicine in Lublin. Informed consent was obtained from the owners prior to clinical investigations and sample collection. All samples were obtained during standard veterinary diagnostic procedures; thus, according to Polish law, the approval by Local Commission for Ethics in Animal Experiments was not required since there was no treatment, including medical, invasive diagnostics, or procedures causing psychological or social discomfort for the participants. Finally, the study was conducted on 43 dogs. As a study group, 15 from the total of 30 diabetic mixed-breed dogs presented by the owner were eligible for the inclusion. The inclusion requirements were: (1) a previous diagnosis of diabetes mellitus; (2) insulin treatment given for at least three months; (3) clinically stable diabetes. Exclusion criteria were any ocular pathology, pancreatitis, adrenal gland hyperactivity, purulent inflammation of the uterus, and bacterial inflammations of the urinary bladder. As a control group, 13 healthy dogs of different breeds were included. They were considered healthy based on physical examination, complete blood cell count, plasma biochemistry profile, and urinalysis. The control group was chosen to be age-matched with the study group. Clinical characteristic of the study groups is shown in Table 1.

Routine biochemical and hematological blood tests and urinalysis were performed for each dog. Each blood sample was collected using a closed vacuum system into a test tube containing EDTA and subjected to hematological analysis in an Exigo Vet analyzer (Boule, Spånga, Sweden). The plasma obtained after centrifugation at 3000 rpm for 15 min at 4 °C was analyzed in a BS-130 automatic biochemical analyzer (Mindray, Shenzhen, China). The chemistry panel included alanine transferase, aspartate aminotransferase, total bilirubin, urea, creatinine, alkaline phosphatase, glucose, albumin, total protein, amylase, γ-glutamyltransferase, and cortisol. For diabetic dogs, glycemic control was estimated using serum fructosamine concentration. Voided midstream urine samples were collected in the morning and basic urinalysis with microscopic sediment analysis was performed on fresh urine samples. A complete ophthalmic examination was performed on all study participants consisting of the slit lamp examination, intraocular pressure measurement, optical coherence tomography, and Schirmer test.

### 2.1. Sample Preparation

For the collection of tear film, Schirmer strips (TearFlo, HUB Pharmaceuticals, 955 236th St NE 1, North Liberty, IA 52317, USA) were placed into the lower sacs of both eyes at 1/3 of the distance of the eyelid from the lateral canthus without anesthesia, as described in similar studies [20,21]. Each collection was performed between 7 and 9 a.m. by the author (DW), using sterile gloves. There is currently no golden standard protocol for the collection of the tear film for their proteomic analysis [22,23]. After holding the strips in place for 5 min, they were removed and transferred to a 1.5-mL Eppendorf tube without any buffer. The strips were then immediately frozen at −80 °C. 3-h extraction of proteins in urea buffer was performed. Extraction was carried out at 4 °C with the addition of protease inhibitor cocktail (Sigma, P8340, Spruce Street, Saint Louis, MO, USA). Strips were removed, and extracts were centrifuged at 5000 rpm for 10 min at 4 °C. Obtained supernatants were collected and stored at −80 °C.

### 2.2. Protein Cleaning and Precipitation

Concentration of proteins was determined by measuring absorbance at 280 nm (MaestroNano Spectrophotometer, Maestrogen, Hsinchu City 30091, Taiwan). The tear fluid containing 150 µg of proteins was placed in a 1.5 mL test tube and topped up with water to 100 µL. To improve the results of electrophoresis and for the protein quantitative precipitation, the ReadyPrep 2-D cleanup kit was applied (Bio-Rad, Warsaw, Poland). Received protein pellets were finally dissolved in 300 µL of the rehydration buffer (ReadyPrep 2-D Starter Kit Rehydration, Bio-Rad, Warsaw, Poland). Obtained solutions were then applied directly to the IPG strips (17 cm, pH 3–10, Bio-Rad, Warsaw, Poland) for the 12 h in-gel rehydration.

The isoelectric focusing was performed in a Hoefer IEF100 device (60 kVh, maximum current: 50 µA per strip). The strips with focused proteins were subsequently equalized in two buffers: the first containing ditiothreitol (2%) and the second containing iodoacetamide (2.5%). The time of each equilibration step was 15 min. A second dimension of the electrophoresis was performed using the 12.5 % polyacrylamide gel. The separation took place in PROTEAN II xi Cell (Bio-Rad, Warsaw, Poland) filled with the Tris/GLY running buffer. The obtained gels were silver stained according to Shevchenko et al. [24] and digitalized using GE healthcare imager. The images of the gels were visually and statistically analyzed in Delta2D software (DECODON, Greifswald, Germany). Delta2D software allowed for the quantitation of spots and creation of protein expression profiles. The featured program is based on warping (correction of positional spot variations and matching images) images of gels to each other leading to so called “fused image”. This “fused image” is a proteome map containing every protein spot obtained on every gel during the whole experiment. After “fused image” creation spots were detected. False negative and false positive protein spots were determined manually. The statistical analysis was made over normalized volume by one-way ANOVA test. The *p*-value in each test was <0.05.

The spots chosen for the further identification were cut out from gels, put into microtubes, rinsed with water and distained. Reduction and alkylation were conducted. These processes were appropriately prepared using ditiothreitol and iodoacetamide solutions. The gel fragments were covered with trypsin. After 20 min, 50 mM ammonium bicarbonate solution was added to the gel spots. The prepared tubes with the gel spots were transferred to the autoclave set for 37 °C for at least 8 h of digestion. After digestion, the extraction of peptide was performed by the three-step extraction in ultrasonic bath (extraction solution composition: acetonitrile:water:trifluoroacetic acid; *v*:*v*:*v* 50:45:5). The received peptide extracts were then concentrated in CentriVap (Labconco, Kansas City, MO, USA) and peptide pellets were created. The pellets were dissolved in 10 µL of trifluoroacetic acid and purified with ZipTip C18 (Merck, Darmstadt, Germany) according to the standard procedure.

### 2.3. MALDI Identification

The appropriate amount of purified peptide extract was put on the AnchorChip MALDI plate with hydrophobic coating and calibrant anchors. The dried peptide spots were covered with the same volume of HCCA (alpha-cyano-4-hydroxycinnamic acid, Bruker, Bremen, Germany) matrix. The peptide calibration standard (Bruker, Bremen, Germany) was spotted on the calibrant spots. Mass spectra were obtained by the Ultraflex III MALDI TOF/TOF (Bruker, Bremen, Germany) spectrometer. All spectra were obtained within the 700–4000 Da *m*/*z* range. Spectra were smoothed and baseline corrected in the flexAnalysis 3.0 software (Bruker, Bremen, Germany). Obtained samples peaks (signal to noise ratio >3) were listed also in the maintained software. After the removal of impurities, lists of peaks positions were transferred to BioTools 3.2 (Bruker, Bremen, Germany). Created lists were then compared by Mascot 2.2 (Matrix Science, London, UK) using the Swiss-Prot database. The chosen taxonomy was “bony vertebrates”, and the maximum error did not exceed 0.3 Da. Possible post-translational modifications are listed in Table 2. According to the Mascot software, a statistically significant score was higher than 56. In the cases of lower scores, the protein samples were analyzed in MS/MS tandem mode.

## 3. Results

Finally, 15 dogs with diabetes mellitus and 13 healthy dogs in the control group were included in proteomic analysis. Diabetic dogs were diagnosed based on clinicopathological variables. They all met the criteria for diabetes mellitus: fasting blood glucose level ≥ 11 mmol/L (200 mg/dL) and glucosuria. Clinical characteristic of the study groups is shown in Table 1.

**Figure 1:**
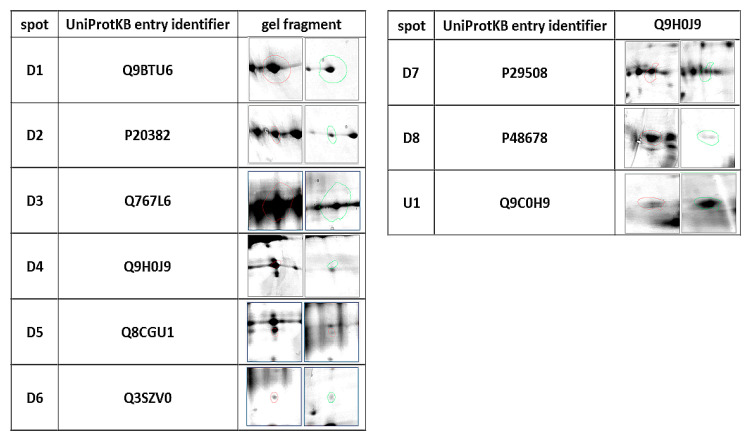
Statistically significant (*p* ≤ 0.05) representatives of 2DE gel spots in the diabetic group compared to control group as revealed by the Delta2D software. The column of spots on the left side are from the control group, and on the right are from the diabetic group.

**Figure 2:**
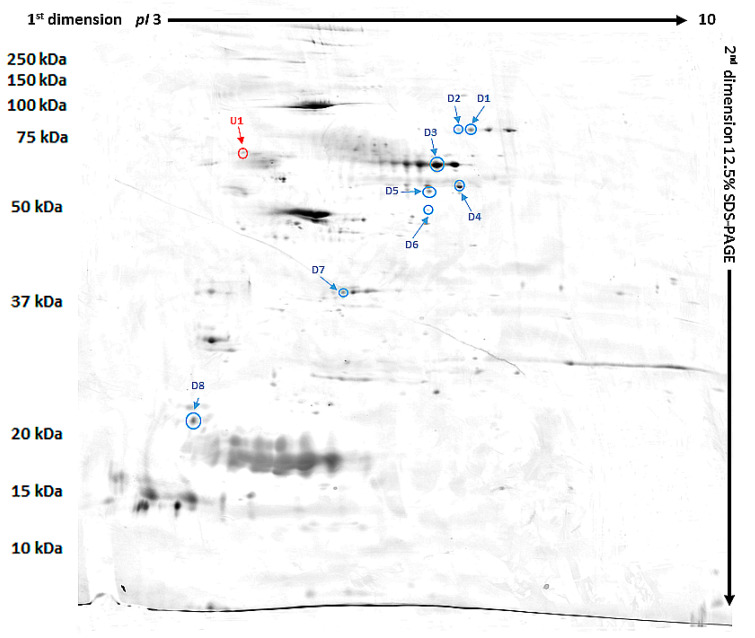
Fused image showing the condensed spot patterns from the experiment. Downregulated proteins are marked in blue while the upregulated proteins are marked in red.

Our study revealed 489 common tear film proteins from the diabetic and control group. From these proteins 14 have shown statistically significant different expression (*p* ≤ 0.05), and so they were excised from the electrophoretic gel. From these 14 tear film proteins, 9 were positively identified by MALDI-TOF MS. Table 2 contains the list of the positively identified proteins, along with their names, genes, and UniProt base accession numbers. With the Delta2D program, eight of the nine proteins were assigned to downregulated, and one of the nine proteins were assigned to upregulated (Table 2, Figure 1). Figure 1 shows representative two-dimensional electrophoresis gel spots of significantly (*p* ≤ 0.05) differentially expressed proteins in the control group versus the diabetes group. Figure 2 shows a fused image of the condensed electrophoretic gels from the whole experiment. Ratio—quotient of the group means of relative spot volumes: volume of a given spot in the control group is the denominator of the ratio parameter (R_t_ > 1.5 overexpression, R_t_ < 0.67 suppression). To further evaluate the nine differentiating proteins, the Panther program (http://www.pantherdb.org) was used to assign them to the appropriate biological process. One protein was identified as upregulated: SRC kinase signaling inhibitor 1 (SRCIN1), and eight proteins were found to be downregulated: phosphatidylinositol-4 kinase type 2 alpha (PI4KIIα), Pro-melanin concentrating hormone (Pro-MCH), Flotillin-1, Protein mono-ADP ribosyltransferase, GRIP and coiled coil domain containing protein 2, tetratricopeptide repeat protein 36, serpin, and Prelamin A/C.

## 4. Discussion

Tear film in diabetes is an easily accessible source of proteins, and was previously investigated in many ways, also using proteomic approaches. Li et al. confirmed tear break-up time (BUT) has lower values in diabetic dry eye patients, and additionally, reported upregulation of proteins related to inflammation, apoptosis and glucometabolic pathways: Annexin A1, neutrophil elastase 2, clusterin, and apolipoprotein A-II [25]. In previous studies, proteomic differences observed only in tear film were insufficient for recognizing diabetic retinopathy with enough sensitivity and specificity. Nevertheless, when these results were combined with retinal photographs evaluation, DR (diabetic retinopathy) could be reliably diagnosed [26,27]. Therefore, we suggest that similar patterns can occur in canine patients.

Gene ontology analysis using the Panther Classification System showed that identified differentiating proteins molecular functions are binding, regulation, and catalytic activity. Protein class analysis categorized proteins as cytoskeletal protein, metabolite interconversion enzyme, or nucleic acid binding protein.

Autoimmune process is thought to play a potentially important role in the etiopathology of canine diabetes [28,29], and therefore, it should be observed in tear film by proteins related to inflammation.

The sole up-regulated protein we were able to identify in diabetic patients was SRC kinase signaling inhibitor 1 (SRCIN1). It is also known as p140 Cas-associated protein (p140CAP), and is responsible for silencing the Src kinases and therefore downregulating multiple signaling pathways like the Ras/extracellular signal-regulated kinase (ERK) pathway, epidermal growth factor receptor (EGFR) pathway [30], or focal adhesion kinase (FAK) pathway [31]. SRCIN1 as a tumor suppressor has been proposed as a potential target in cancer studies [32].

Src family kinases (SFKs) have been recently reported as a potent mediators of the VEGF pathway. VEGF is a major protein secreted by the ischemic retina due to the blood-retinal-barrier (BRB) breakdown in diabetic patients. Eventually, it leads to the formation of the retinal neovascularization. These vessels, due to their pathological anatomy and lack of pericytes, tend to leak, causing retinal edema and hemorrhages. Anti-VEGF intravitreal injections are currently a treatment of choice in diabetic retinopathy. Thus, up-regulation of the Src kinase inhibitor may reflect the VEGF/SRC kinase signaling axis activation in the diabetic group.

Prelamin-A/C is a precursor protein for lamin A and lamin C, major constituents of nuclear lamina [33]. They are essential for maintaining the cellular homeostasis, but point mutations in encoding LMNA genes, and excessive aggregation of farnesylated lamin A leads to premature cell senescence, causing a spectrum of diseases called laminopathies, including Hitchinson-Gilford progeria, a severe premature aging disease. One study has shown that heterozygous rare missense mutations in LMNA gene encoding nuclear lamin A/C in diabetes patients are associated with severe metabolic alterations, such as hypertriglyceridemia and insulin resistance [34]. Disturbances in prelamin A/C expression and its further final products, lamin A and C, cause significant oxidative stress, mitochondrial dysfunction and eventually lead to apoptosis [35,36]. Prelamin A/C was downregulated in the tear film of our patients, which may either be a result of its insufficient expression, or excessive usage in the course of lamins A and C production. Both situations are connected to inflammation, increased oxidative stress, and apoptosis observed in canine diabetes etiopathology. Alongside, Panther GO bio software linked Prelamin-A/C with the FAS pathway that plays a fundamental role in the regulation of the immune system through apoptosis. This, accompanied by the involvement of SRCIN1 in also apoptosis related ERK pathway, shows some evidence that those two proteins may be involved in cell death related to diabetes.

Flotillin-1 belongs to the family of flotillins, transporting proteins forming cellular membrane rafts [37]. Those rafts take an important part in the formation of caveolae: indentations of the mammalian cellular membrane, highly expressed especially in endothelial cells and adipocytes. Caveolae serve multiple functions, including endocytosis and signal transduction [38,39]. It was demonstrated in Type 2 diabetic mice that low expression of flotillin-1 in liver may contribute to metabolic dislipoproteinemia [40]. Interestingly, Flotillin-1 mediates endocytosis linked to Fyn kinase, which is a member of Src kinases [41]. This links the downregulation of Flotillin-1 with upregulation of SRCIN1. The coexistence of those proteins may suggest Src kinases pathway impairment in the course of canine diabetes.

Pro-Melanin-concentrating hormone (Pro-MCH) is a prohormone of melanin-concentrating hormone (MCH), tightly connected to obesity and increased food intake in animals [42,43]. MCH is a neuropeptide expressed mainly in the hypothalamus. It acts through two major receptors: MCHR1 and MCHR2, and as MCHR1 is common in mammals, MCHR2 is found only in certain species including dogs [42,44,45]. MCH is activated by palatable food intake and promotes further consumption and energy conservation [42]. This mechanism can be connected to diabetics frequent eating disorders, which promotes further disease development. In our study pro-MCH was the downregulated protein in tear film. Like in Prelamin-A/C, it is hard to say whether this is due to its excessive use and potentially elevated levels of MCH, or a general decrease in expression MCH pathway. Anyhow, lack of MCHR2 expression in rats, mice, and rabbits—the most popular animal models of diabetes—and its presence only in dogs, ferrets and rhesus monkeys [45] supports another idea of using the canine animal model for a proper resemblance of human diabetes.

Phosphatidylinositol-4 kinase type 2 alpha (PI4KIIα) is a crucial kinase in the exo-endocytic cycle of synaptic vesicle, being both membrane protein cargo and an enzymatic regulator of adaptor function [46,47]. It is also involved in exocytosis of secretory granules containing insulin in pancreatic cells [48]. Interestingly, mice lacking one of the like PI4KIIα enzymes, phosphatidylinositol 5-phosphate 4-kinase β, showed increased insulin sensitivity with normal adiposity [49]. We found PI4KIIα to be downregulated in tear film of diabetic patients. As the hallmark of canine diabetes is impaired insulin excretion, lowered PI4KIIα levels fit into this pattern, along with other identified proteins being heavily involved in exo- endocytosis mechanisms, like Flotillin-1, or GRIP and coiled coil domain containing protein 2.

Protein mono-ADP ribosyltransferase (PARP12) is a regulatory cytoplasmic protein involved in response to stress induced by unfolded protein response (UPR) of the endoplasmic reticulum (ER) [50]. Usually ADP-ribosylation effects in inactivation of affected proteins, being a reversible post-translational modification, and is related to multiple, both physiological and pathological cellular processes [51].

Poly (ADP-ribose) polymerases (PARP) play an important role in the pathogenesis of diabetes and its complications. Their activation contributes to the injury of β-cells and endothelium. Hyperglycemia induced PARP activation affects predominantly the retinal vasculature [52]. It was shown that protein mono-ADP-ribosylation might be initiated by diabetes and capable of causing peripheral diabetic neuropathy. In rats with diabetes induced with alloxan ADP-ribosyltransferase, activity in the cell body of peripheral and retina neurons was higher than normal increasing the level of mono-ADP-ribosylation of selected proteins leading to diabetic neuropathy [53]. Pharmacologic inhibition of PARP attenuates some features of the β-cell death, cardiovascular injury, and some other complications. PARP activation is present in individuals belonging to the group being at risk of developing diabetes and so it is suggested to be a potential early marker of this disease [52].

GRIP and coiled coil domain containing protein 2 is a part of coiled-coil proteins associated with Golgi membranes called golgins, that interact with Golgi membranes via GRIP domain. In general, those proteins are responsible for proper vesicle formation, docking, and transport, playing an important role in intracellular signaling [54,55,56,57]. Despite the ubiquitous expression of those proteins in mammalian cells, there is currently no evident link between their dysregulation and diabetes. In our study, we found GCC domains containing protein 2 to be downregulated in diabetic patients. It stays in line with other proteins that are also involved in intracellular signaling, probably acting in a similar albeit complementary way.

Tetratricopeptide repeat protein 36 (TPR36) TPR motif is a protein–protein interaction module involved in cell-cycle, transcription, and protein transport [58]. TPR36 was reported to be expressed mainly in liver, kidney, and testis in mice [59].

Serpin B3 is a member of the family of evolutionary conserved serine protease inhibitors, which exert various biological functions. Serpin B3 is usually linked with immunity and cell death mechanisms, but its exact physiological function remains elusive. It is excreted by epithelial, endothelial, and blood cells. Its expression is known to be induced by tumor necrosis factor α (TNF-α) [60]. In one proteomic study that investigated the mechanisms underlying delaying wound healing in diabetes it was revealed that Serpin B3 was strongly upregulated in groups of patients with rapidly healing vs groups with non-healing wounds [61]. A transgenic mouse model with α1-antitrypsin promoter-driven overexpression of SERPINB3 was proposed to test Serpin B3 relevance in in vivo conditions. This model showed no effect of SERPINB3 overexpression on wound healing in both non-diabetic or diabetic mice with or without hindlimb ischemia. In an independent validation cohort of 47 patients, high serpin B3 protein content was confirmed as a biomarker of healing improvement [61].

## 5. Conclusions

We believe that this is the first study to evaluate the proteomic composition of tear film in canine diabetes. It is worth noticing that a significant number of identified proteins play a role in intracellular signaling: vesicle formation, bonding, and transport through membranes. This may suggest that the first signs of diabetic cellular impairment may be recognized in tear film composition before any clinical signs occur. Further studies involving more animals and a more targeted approach may be an option for the upcoming research. In future, animal models of diabetes combined with tear film proteomic analysis may be a viable option for better understanding and monitoring of the disease.

## Figures and Tables

**Table 1:** Clinical characteristic of the study subjects.

Variable	Healthy Subject n = 13	Patients with Diabetes n = 15
Age, Y		
Mean (±SD) Range	10.8 ± 1.8 8–13	10.3 ± 2.8 7–16
Sex		
Male Female	7 6	7 8
Weight, kg		
Mean (±SD) Range	23.1 ± 7.7 10–37	13.9 ± 8.6 5.4–38
Duration of diabetes, y		
Mean (±SD) Duration	0 0	1.2 ± 0.65 0.5–2
Blood glucose, mmol/L		
Mean (±SD) Range	5.9 ± 0.2 5.6–6.2	17.8 ± 6 8.3–29.7
Retinopathy	0	5
Cataract	0	9

**Table 2:** Significantly (*p* ≤ 0.05) differentially expressed proteins in diabetic dogs as identified by MALDI-TOF MS.

ID	Protein	Accession Number (UniProtKB)	Species	Score	Match	MW (kDa) *	pI *	Modif.	Seq. Cov (%)	Rt **	*p* Value
D1	Phosphatidylinositol 4-kinase type 2-alpha	Q9BTU6	*H. sapiens*	74	11	54.388	8.51	C, O	26	0.437	0.032
D2	Pro-MCH	P20382	*H. sapiens*	75	6	18.781	6.74	C, O	38	0.350	0.04
D3	Flotillin-1	Q767L6	*Sus scrofa*	65	10	47.558	7.66	C, A, O	24	0.491	0.03
D4	Protein mono-ADP ribosyltransferase	Q9H0J9	*H. sapiens*	66	11	80.496	8.84	C, A, O	23	0.403	0.03
D5	GRIP and coiled-coil domain-containing protein 2	Q8CGU1	*M. musculus*	90	21	195.294	5.07	C, O	14	0.252	0.03
D6	Tetratricopeptide repeat protein 36	Q3SZV0	*B. taurus*	81	11	20.660	5.09	C, O	40	0.232	0.02
D7	Serpin B3	P29508	*H. sapiens*	71	14	44.594	6.35	C	38	0.530	0.04
D8	Prelamin A/C,	P48678	*M. musculus*	72	18	74.478	6.54	C, O	31	0.170	0.001
U1	SRC kinase signaling inhibitor 1	Q9C0H9	*H. Sapiens*	66	15	127.198	9.39	C, O	15%	2.289	0.03

Abbreviations: C—carbamidomethylation of cysteine; O—oxidation of methionine; A—acetylation of protein N-term. Listed molecular weights and * pI values correspond to the MASCOT Search Result, ** Rt (Ratio) quotient of the group means of relative spot volumes; volume of a given spot in control group is the denominator of the ratio parameter.
